# Loss of zebrafish *dzip1* results in inappropriate recruitment of periocular mesenchyme to the optic fissure and ocular coloboma

**DOI:** 10.1371/journal.pone.0265327

**Published:** 2022-03-14

**Authors:** Sri Pratima Nandamuri, Sarah Lusk, Kristen M. Kwan

**Affiliations:** Department of Human Genetics, University of Utah, Salt Lake City, UT, United States of America; Iveric Bio, UNITED STATES

## Abstract

Cilia are essential for the development and function of many different tissues. Although cilia machinery is crucial in the eye for photoreceptor development and function, a role for cilia in early eye development and morphogenesis is still somewhat unclear: many zebrafish cilia mutants retain cilia at early stages due to maternal deposition of cilia components. An eye phenotype has been described in the mouse *Arl13* mutant, however, zebrafish *arl13b* is maternally deposited, and an early role for cilia proteins has not been tested in zebrafish eye development. Here we use the zebrafish *dzip1* mutant, which exhibits a loss of cilia throughout stages of early eye development, to examine eye development and morphogenesis. We find that in *dzip1* mutants, initial formation of the optic cup proceeds normally, however, the optic fissure subsequently fails to close and embryos develop the structural eye malformation ocular coloboma. Further, neural crest cells, which are implicated in optic fissure closure, do not populate the optic fissure correctly, suggesting that their inappropriate localization may be the underlying cause of coloboma. Overall, our results indicate a role for *dzip1* in proper neural crest localization in the optic fissure and optic fissure closure.

## Introduction

Vertebrate eye development, comprising numerous conserved gene regulatory networks and morphogenetic processes, begins with the emergence, or evagination, of optic vesicles from the ventral forebrain. The newly formed optic vesicles then undergo a series of specification and patterning events that are integrated with changes in tissue shape in order to generate the optic cup, in which neural retina and retinal pigment epithelium enwrap the lens [1–8]. Formation of a precise and stereotyped eye structure is crucial for visual function, and morphological aberrations of the eye are a common cause of human visual impairments.

One such visual impairment condition is ocular coloboma, which accounts for ~10% of pediatric blindness worldwide. Coloboma is caused by defective development and morphogenesis of a specific structure in the eye, the optic fissure. The optic fissure initially forms during the process of eye morphogenesis, as two closely apposed tissue margins appear at the ventral side of the optic cup. As retinal neurogenesis proceeds, the optic fissure undergoes tissue closure, fusing along its proximodistal length to generate a seamless channel through which the hyaloid vasculature enters the eye and the retinal ganglion cell axons exit and project to the brain [9–15]. Human genetic and model organism studies of coloboma have uncovered many genes required for optic fissure development, including specific cilia and Hedgehog (Hh) signaling components [16–20].

Cilia play crucial roles in the development of many different organ systems, with primary cilia playing a well-documented role in Hedgehog signaling [21–25]. In the eye, human ciliopathies can provide some insight: although impairments to cilia are most often associated with retinal dystrophy, loss of photoreceptor structure and function, and retinal dysfunction and degeneration [26–29], certain cilia gene mutations associated with COACH/Joubert syndrome can result in distinct clinical presentations, including coloboma [30–33]. However, roles for cilia earlier in eye development and morphogenesis are not well-described. It was shown that a mouse knockout of the small GTPase *Arl13b* resulted in disrupted cilia and dramatic eye morphogenesis phenotypes that were a result of mis-regulated Hh signaling [34, 35], although arl13b can function in Hh signaling from outside of the cilium [36]. In zebrafish, many studies of known cilia mutants have not identified early phenotypes, in part because maternal deposition of cilia factors may compensate for early developmental processes. For example, *arl13b* is maternally expressed in zebrafish; *arl13b* mutants exhibit shorter photoreceptor outer segments and progressive photoreceptor degeneration but no early eye phenotype [37, 38]. The zebrafish *MZift88* mutant exhibits a loss of cilia from early stages, however, the eye was not examined in detail [39], and the stages of eye morphogenesis associated with coloboma have not been closely examined in zebrafish cilia mutants.

We sought to examine eye development and morphogenesis in a mutant that might remove cilia starting from early stages. The *dzip1* mutant (*iguana*^*ts294e*^) was isolated for several phenotypes, including midline and retinotectal axon pathfinding defects [40–44]. Some defects were recognized to be reminiscent of alterations in Hedgehog (Hh) signaling, and it was established that the gene is crucial for proper Hh signal transduction in developing muscle and neural tube [45, 46], although the eye was not closely examined. Cloning of the *iguana* locus revealed that the affected gene is *dzip1*, which encodes a zinc finger and coiled coil domain protein [45, 46]. The dzip1 protein has been reported to localize to the primary cilia basal body and the ciliary transition zone, and it is required for primary cilia, but not motile cilia, formation [47–49]. Similar to arl13b, dzip1 functions both in cilia formation and also has a cilia-independent function in Hh signaling by controlling Gli turnover kinetics; dzip1 can also function in the centrosome [50–53], In both Drosophila and zebrafish *dzip1* mutants, docking of the basal body occurs, but without any axonemal growth [48, 49]. Strikingly, in zebrafish, no primary cilia were observed as early as 10 hpf [49], making this a intriguing model to examine cilia, Hedgehog signaling, and centrosomes in early eye development.

Here, we find that *dzip1* mutant eyes lack cilia throughout stages of early development, and embryos exhibit coloboma with incomplete penetrance. We find that early eye formation proceeds normally, and the phenotype arises during optic fissure closure. We examined specific cell populations and processes required for optic fissure closure, and found that neural crest cells, but not endothelial cells, exhibit defects in initial localization to the optic fissure at 24 hpf. Further, we asked how Hh signaling is affected in the eye when cilia are lost in the *dzip1* mutant. Somewhat surprisingly, we found variable effects on induction of Hh target genes in the early eye: *pax2a*, is unaffected in the *dzip1* mutant, but *ptch2* is lost. In contrast, retinal ganglion cell differentiation, another Hh-dependent process, is impaired in the *dzip1* mutant. These results suggest that loss of cilia in the *dzip1* mutant can lead to coloboma, possibly through effects on the neural crest.

## Materials and methods

### Zebrafish husbandry & mutant/transgenic lines

All zebrafish (*Danio rerio*) husbandry was performed at the Centralized Zebrafish Animal Resource facility (CZAR) at the University of Utah. Standard operating conditions were followed in accordance with approved University of Utah Institutional Animal Care and Use Committee (IACUC) Protocol #21–01007. Zebrafish embryos (Tu strain) were collected within 10 minutes of fertilization, raised at 28.5°–30°C, and staged according to time post fertilization and morphology [54].

*dzip1* (*iguana*^*ts294e*^) mutants contain a point mutation (C/T) resulting in a transcript with a premature stop codon: Gln>Stop at position 371 [45, 46]. *dzip1*^*-/-*^ are not viable as adults and were maintained as *dzip1*^*+/-*^. Transgenic alleles used in the experiments were *Tg(bactin2*:*Arl13b-GFP)*^*hsc5Tg*^ [55], *Tg(bactin2*:*EGFP-CAAX)*^*z200*^ [56], *Tg(sox10*:*memRFP)*^*vu234*^ [57] and *Tg(kdrl*:*ras-mCherry)*^*s896Tg*^ [58].

### Genotyping

Genomic DNA was extracted by incubating single embryos or adult tail fins in 0.05M NaOH at 95°C for 30 minutes, followed by neutralization with 1M Tris (pH 8.0). The *dzip1* locus was genotyped using High Resolution Melt Analysis (HRMA) protocol [59] with the following primers: Forward: 5’ GTCAAAGCCGCCAACAATAACA 3’ and Reverse: 5’ AAAATCCACAAACCTTCGCGCT 3’. These primers generate a 48 bp amplicon; homozygous wild type and mutant duplexes melt at 81°C and 82°C, respectively.

### Coloboma scoring

Embryos generated from *dzip1*^*+/-*^ incrosses were screened individually for coloboma at 52-55hpf using an Olympus SZX16 stereomicroscope. Embryos were scored as positive for coloboma if the eyes displayed an area of hypopigmentation in the region of the optic nerve head on the ventral side. All genetic experiments were scored blinded to genotype. Following scoring, embryos were individually genotyped as described above.

### Immunofluorescence

Embryos were raised until the relevant stage (24, 36 or 48 hpf) and fixed in 4% paraformaldehyde (EMS, 15710) either overnight at 4°C or for 2 hours at room temperature. Embryos older than 24 hpf were maintained in PTU+E3 (0.003% 1-phenyl-2-thiourea (P7629, Sigma-Aldrich), added around 21–22 hpf to prevent pigment formation. Following permeabilization in PBST (PBS+ 0.5% Triton X-100) and blocking in PBST+2% bovine serum albumin for an hour at room temperature, embryos were incubated in primary antibodies overnight at 4°C. Embryos were then washed in PBST, and secondary antibodies diluted in blocking solution were added for 3–4 hours at room temperature. Nuclei were detected by incubation with 1 μM TO-PRO-3 iodide (T3605, Invitrogen). Prior to imaging, embryos were cleared overnight at 4°C in 70% glycerol (in PBS).

Primary antibodies and their concentrations are as follows: anti-Activated Caspase-3 (1:700, #559565, BD Pharmingen), anti-Phospho-Histone-H3 (1:500, ab14955, Abcam), anti-Laminin1 (1:500, L9393, Sigma-Aldrich), anti-Zn-5 (1:500, ZDB-ATB-081002-19, Zebrafish International Resource Center), anti-Pax2a (1:200; Genetex, GTX128127), and anti-Islet1/2 (1:100 concentrate; DSHB 39.4D5). All secondary antibodies were used at a concentration of 1:200 and included: Alexa Fluor 488 goat anti-mouse (A-11001, Invitrogen) and Alexa Fluor 488 goat anti-rabbit (A-11008, Invitrogen).

### HCR fluorescence in situ hybridization for ptch2

RNA fluorescent in situ hybridization using hybridization chain reaction (HCR) was performed on whole mount zebrafish embryos at 24 hpf. Probes and HCR reagents were purchased from Molecular Instruments. Our protocol was based off of the publicly available protocol, “HCR RNA-FISH protocol for whole-mount zebrafish embryos and larvae” (Molecular Instruments; [60]); our modifications include smaller reagent volumes but maintain the same concentration of probe and hairpins. Hairpins were conjugated to Alexa Fluor 647.

### Imaging

For imaging, both live and fixed embryos were embedded in 1.6% low melting agarose (in E3 or PBS) in Pelco glass-bottom dishes (14027, Ted Pella). E3 or PBS was overlaid in the dishes to prevent evaporation. All imaging was performed using a 40X water immersion objective (1.2 NA) on the Zeiss LSM880 laser-scanning confocal microscope with the following parameters: 2.1 μm z-step, 512x512 frame size, 0.7 zoom. After acquiring imaging datasets, embryos were de-embedded and genotyped.

### Image analysis

Z-stacks were processed using FIJI [61]. Three-dimensional rendering was executed in FluoRender [62]. For lateral views, the ectoderm was digitally erased in FIJI to permit visualization of the eye and lens and quantification of the data. For all image quantification, measurements were obtained blinded to genotype.

### Image quantification: Optic fissure opening angle

Three-dimensional data sets of live embryos labeled for cell membranes (EGFP-CAAX) were oriented laterally in FluoRender. With the lateral cutaway tool, the 3D rendering was cut along the proximal-distal axis to the lens midpoint. This orientation was captured in FluoRender and saved as a TIFF image file. The optic fissure opening angle was measured using the angle tool in FIJI. As schematized in Fig 2M, the vertex was positioned at the center of the lens with the rays of the angle extending to each of the optic fissure margins.

### Image quantification: Activated caspase-3 immunofluorescence

As schematized in Fig 3G, a rectangular region of interest (ROI), the width of which matched the width of the lens, was defined for each embryo. All activated caspase-3 labeled nuclei within this ROI throughout the depth of the optic fissure within the eye were manually counted in FIJI by stepping through the volume data slice by slice. Labeled cells in the lens were excluded.

### Image quantification: Phospho-histone H3 immunofluorescence

As schematized in Fig 4G, with the eye viewed in a lateral orientation, a rectangular region of interest (ROI), the width of which matched the width of the lens, was defined for each embryo. All phospho-histone-H3 labeled cells within this ROI throughout the depth of the optic fissure within the eye were manually counted in FIJI by stepping through the z-stack slice by slice. Labeled cells in the lens were not counted.

### Image quantification: Laminin fluorescence

3-dimensional datasets were acquired of laterally oriented embryos. For each embryo, a range of slices encompassing the lens were chosen; the most distal slice was the one where the lens was just starting to be visible and the proximal slice was the one behind the lens. Within this range, three single optical sections were selected from different depths along the proximal-distal axis. The three optical sections (proximal, middle, and distal) were equidistant from each other and bisected the lens. Quantification of laminin fluorescence was only performed on the most proximal section (indicated by asterisk in the schematic in Fig 5). In the proximal z-slice, a rectangular ROI (area = 400 μm^2^) oriented along dorso-ventral axis encompassing optic fissure margins was defined (schematized in 5M). Laminin fluorescence intensity within the ROI was calculated as the mean gray value using the Measure function in FIJI. Similarly, the mean gray value of laminin fluorescence in the same ROI placed on the nasal boundary of the eye next to the olfactory placode was also measured in the same proximal slice; this is a region in which laminin fluorescence would not be expected to vary between embryos and over developmental time. Fluorescence intensity of laminin at the optic fissure was normalized to the fluorescence intensity of laminin at the nasal boundary of the eye.

### Image quantification: Tg(sox10:memRFP) positive neural crest cells

Three-dimensional data sets of laterally oriented embryos labeled for neural crest cells *Tg(sox10*:*memRFP)* were acquired. As schematized in Fig 6G, transgene-positive cells specifically in the optic fissure within the eye were manually counted by stepping through the z-stack slice by slice in FIJI. Transgene-positive cells located in the fissure and in contact with the retinal margins were included.

### Image quantification: Hyaloid vessel width

3-dimensional imaging datasets of laterally oriented embryos were acquired. For each embryo, a maximum intensity projection (MIP) of the vasculature channel (*kdrl*:*ras-mCherry*) was created in FIJI. The range of slices used for the MIP was determined such that the most distal slice was the one where the lens was just visible and the most proximal slice was behind the lens. From the MIP, the width of the widest portion of the blood vessel passing through the optic fissure was measured using the length tool in FIJI.

### Image quantification: Superficial ocular vasculature

3-dimensional imaging datasets were laterally oriented in FluoRender, and the number of ocular vessels in the dorsal hemisphere of the eye were counted.

### Image quantification: Pax2a-positive domain

3-dimensional datasets were acquired of dorsally oriented embryos. For each embryo, the eye was oriented laterally in FluoRender. Using the cutaway tool, the 3-dimensional volume was cut to the lens midpoint. This image was captured in FluoRender and quantified in FIJI using the angle measurement (schematized in Fig 8C): the vertex of the angle was placed at the center of the lens and the rays of the angle were drawn to encompass the entire domain (nasal to temporal) of Pax2a-positive staining.

### Image quantification: Pax2a fluorescence intensity

3-dimensional datasets were acquired of dorsally oriented embryos. For each embryo, a maximum intensity projection (MIP) was generated in FIJI. As schematized in Fig 8D, an elliptical ROI (area = 7000 μm^2^) was drawn surrounding the optic fissure and stalk area. The fluorescence intensity of pax2a staining within the ROI was calculated as the mean gray value using the Measure function in FIJI. The mean gray value of an identically sized elliptical region at the dorsal eye was also measured as a control region. The mean fluorescence intensity of pax2a in the optic fissure and stalk region was normalized to the region in the dorsal eye.

### Image quantification: ptch2 HCR in situ hybridization fluorescence intensity

3-dimensional datasets were acquired of laterally oriented embryos. For each embryo, a maximum intensity projection (MIP) for the entire volume data was generated in FIJI. As shown in Fig 8E and 8F, an elliptical ROI was drawn surrounding the optic fissure and stalk area. The fluorescence intensity of *ptch2* staining within the ROI was calculated as the mean gray value using the Measure function in FIJI. The mean gray value of an identically sized elliptical region in the dorsal eye was also measured as a control region. The mean fluorescence intensity of *ptch2* mRNA staining in the optic fissure and stalk region was normalized to the region in the dorsal eye.

### Image quantification: Retinal ganglion cell (zn-5) fluorescence

3-dimensional datasets were acquired of laterally oriented embryos. For each embryo, a maximum intensity projection (MIP) was generated in FIJI. The range of slices used for the MIP was determined such that the most distal slice was the one where the lens was just visible and the most proximal slice was just behind the lens. As schematized in Fig 8K, an elliptical ROI (area = 18000 μm^2^) surrounding the retinal ganglion cell region was defined. The center of the elliptical ROI overlapped the center of lens. The fluorescence intensity of zn-5 staining within the ROI was calculated as the mean gray value using the Measure function in FIJI. Similarly, the mean gray value of zn-5 in a rectangular ROI (area = 3300 μm^2^) placed in the brain, dorsal to the eye, was also measured. The mean fluorescence intensity of zn-5 in the eye was normalized to the fluorescence intensity of zn-5 in the region outside the eye.

### Image quantification: Islet1-positive RGC fluorescence intensity

3-dimensional datasets were acquired of laterally oriented embryos at 72 hpf. For each embryo, a maximum intensity projection (MIP) for the entire volume data was generated in FIJI. The RGC layer was identified based on its stereotypical position in the retina. As schematized in Fig 8N, a square ROI (area = 1100 μm^2^) was placed at nasal, temporal, and dorsal positions in the RGC layer, and fluorescence intensity was measured as the mean gray value using the Measure function in FIJI. These three measurements were averaged to provide an average fluorescence intensity throughout the RGC layer. The ventral side of the RGC layer was not used in order to avoid aberrations that might be caused by the coloboma phenotype. The mean fluorescence intensity was also measured in a control region in the brain, dorsal to the eye using the same square ROI (area = 1100 μm^2^). The mean fluorescence intensity in the RGC layer (the average of the nasal, temporal, and dorsal measurements) was normalized to the fluorescence intensity in the control region.

### Image quantification: Islet1/2-positive RGC layer width

3-dimensional datasets were acquired of laterally oriented embryos at 72 hpf. For each embryo, a maximum intensity projection (MIP) for the entire volume data was generated in FIJI. The RGC layer was identified based on its stereotypical position in the retina. As schematized in Fig 8O, the width of the RGC layer was measured at three positions (nasal, temporal, and dorsal) by drawing a line across the layer and using the Measure function in FIJI. These three positions were averaged for each embryo to provide an average RGC layer width per embryo.

### Statistical analysis

Box and whisker plots were generated using the ggplot2 package in R. The lower and upper hinges correspond to the first and third quartiles. The line inside the box represents the median. The upper whisker extends from the upper hinge to the highest value within (1.5×IQR), where IQR is the inter-quartile range. The lower whisker extends from the lower hinge to the lowest value within (1.5×IQR). Data points outside of the ends of the whiskers are outliers. For comparison between the groups, *P*-values were calculated using a two-sample t-test that takes into consideration unequal sample sizes and/or unequal variances. A 95% confidence interval (*P*<0.05) was chosen to indicate statistical significance.

## Results

### Loss of dzip1 leads to loss of cilia in the developing eye and coloboma

The molecular machinery underlying cilia formation and function has been examined in the eye, particularly in photoreceptor structure and function [26–28]. Despite a clear role for cilia in these important visual processes, functions in early eye development have not been evaluated. A role for cilia in eye development and morphogenesis is suggested due to the association of certain ciliopathies with coloboma, a visual impairment condition due to disrupted development of the optic fissure. Further, in zebrafish, many cilia mutants that have been studied have not been reported to exhibit defects in early eye development, however, functional roles may be masked due to maternal deposition of wild type RNAs and proteins. To avoid this, we took advantage of the *dzip1* mutant [45, 46, 49], which displays a loss of cilia as early as 10 hpf, a stage when optic vesicle evagination is commencing. Eye development was not previously evaluated in the *dzip1* mutant, therefore, this mutant provides a unique opportunity to examine its functions in cilia formation and Hh signaling in early eye development, prior to retinal neurogenesis and photoreceptor development.

Primary cilia in the eye were not previously analyzed in the *dzip1* mutant, therefore we first sought to confirm that the *dzip1* mutant does indeed display a loss of cilia during eye development. *dzip1* heterozygous adults were crossed with the *Tg(bactin2*:*arl13b-GFP)* line [55], in which the small GTPase arl13b, which is localized to the ciliary axonemal membrane, is fused to GFP and expressed ubiquitously, thereby facilitating visualization of cilia in live embryos.

**Fig 1:**
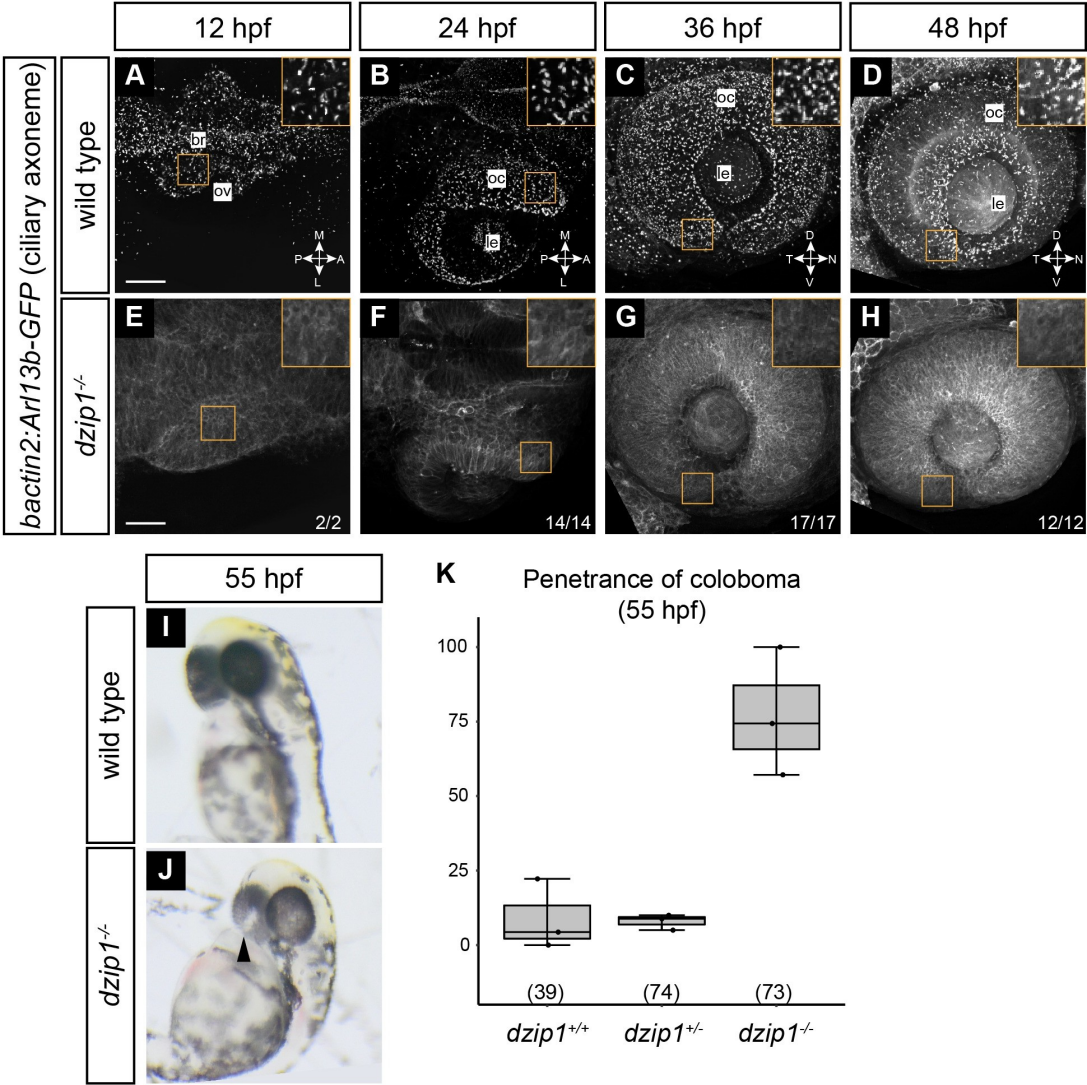
*dzip1* mutants have no cilia in the eye and display coloboma. (A-H) Wild type (A-D) and *dzip1*^*ts294e*^ mutants (E-H) visualized for cilia (*Tg(bactin2*:*Arl13b-GFP)*) at 12 hpf (A, E), 24 hpf (B, F), 36 hpf (C, G), and 48 hpf (D, H). A, B, E, and F are dorsal views of 3-dimensional renderings; while C, D, G, and H are lateral views of 3-dimensional renderings. (A-D) GFP-positive cilia are visualized as distinct puncta present throughout the lens, eye, and embryo midline (A, *magenta arrowhead*). (E-H) Cilia are completely absent in *dzip1*^*-\-*^ embryos. n (fraction of *dzip1*^*-/-*^ embryos showing complete absence of cilia) is shown at the bottom right of each panel. Insets show zoomed views for each timepoint and genotype. (I-K) *dzip1*^*-/-*^ mutants display coloboma. (I) Wildtype embryo at ~55 hpf; the eye is evenly pigmented; there is no coloboma. (J) *dzip1*^*ts294e*^ mutant embryo, ~55 hpf; coloboma is visible as a hypopigmented region at the back of the eye (*arrowhead*). (K) Penetrance of the coloboma phenotype at 55 hpf. n (embryos) for each genotype shown at the base of the graph. Scale bar: 50 μm. M, medial; L, lateral; A, anterior; P, posterior; D, dorsal; V, ventral; N, nasal; T, temporal; le, lens; oc, optic cup.

At 12, 24, 36, and 48 hpf, in wild type embryos, the arl13b-GFP transgene robustly labeled cilia throughout the eye (Fig 1A–1D). In contrast, we found diffuse fluorescence with no apparent punctate ciliary localization in *dzip1* mutants (Fig 1E–1H), indicating a loss of cilia. For Fig 1A–1H, insets provide a zoomed view of the presence or absence of cilia. Therefore, the *dzip1* mutation does indeed lead to loss of cilia in the eye throughout the timeline of early eye development. During these experiments, we noticed that *dzip1* mutants exhibit bilateral coloboma at 55 hpf (Fig 1I and 1J), as characterized by the presence of a hypopigmented region at the back of both eyes near the optic nerve head. This phenotype was not 100% penetrant (Fig 1K; 77.17±21.57% *dzip1* mutants with coloboma), and few heterozygous or wild type embryos presented with coloboma (wild type 8.86±11.78%; heterozygous 7.94±2.61%). We therefore sought to determine the basis for the coloboma phenotype in the *dzip1* mutant.

### Optic fissure closure, but not optic fissure formation, is defective in dzip1 mutants

Coloboma arises due to disrupted development of the optic fissure, a transient structure at the ventral side of the eye. The optic fissure initially forms during optic cup morphogenesis by 24 hpf, as two closely apposed tissue margins appear at the ventral side of the embryonic eye and optic stalk. Subsequently, the optic fissure closes: the tissue margins undergo fusion, during which the basal lamina lining each tissue margin are broken down, and cells from each side make direct contact and rearrange to generate a seamless retina and RPE [9, 11, 12, 15, 56].

**Fig 2:**
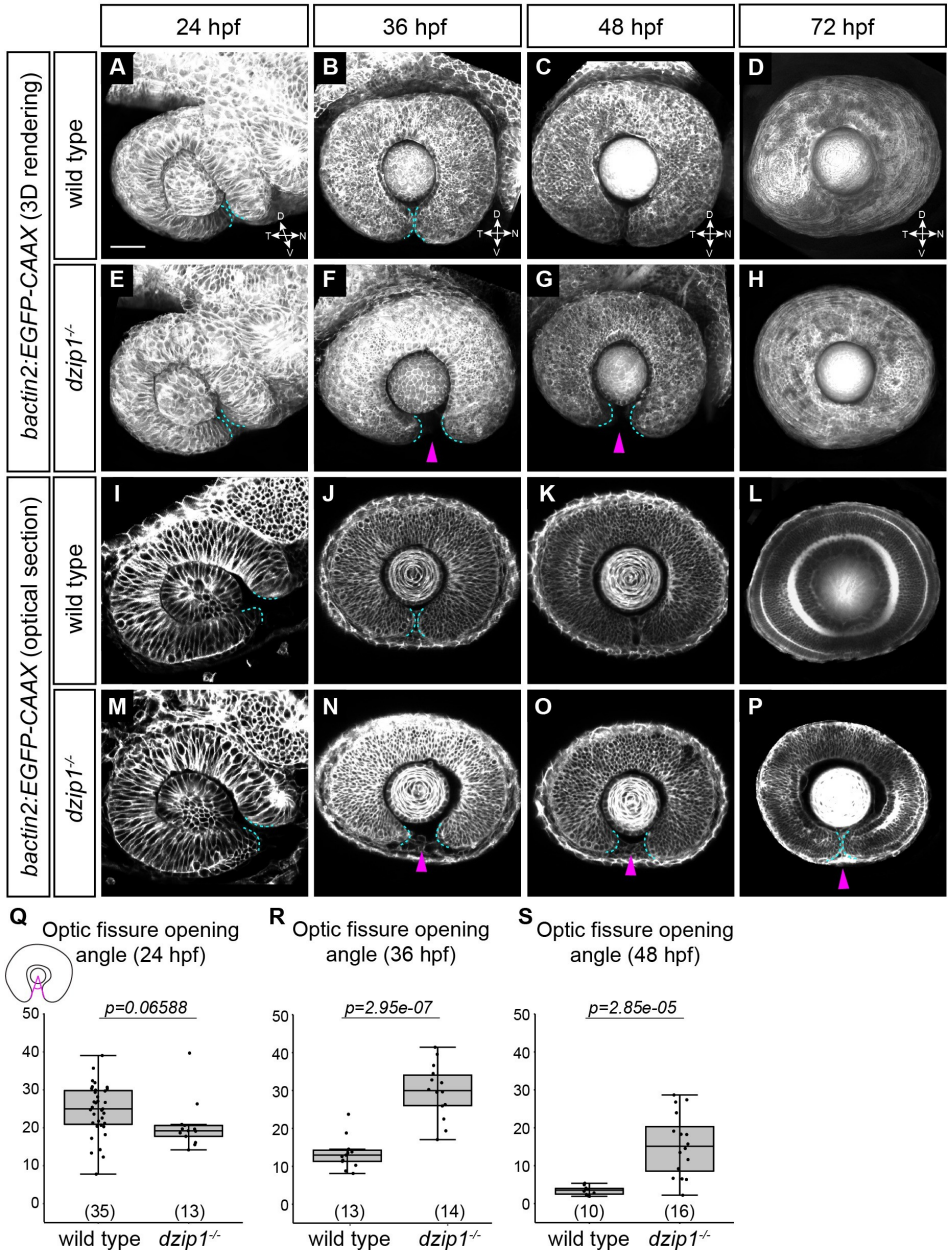
*dzip1* mutants show defects in optic fissure closure but not optic fissure formation. (A-P) Wild type (A-D, I-L) and *dzip1*^*ts294e*^ mutants (E-H, M-P) visualized for membranes (*Tg(bactin2*:*EGFP-CAAX)*) at 24 hpf (A, E, I, M), 36 hpf (B, F, J, N), 48 hpf (C, G, K, O), and 72 hpf (D, H, L, P). (A-H) Images are lateral views of 3-dimensional renderings. (I-P) Images are lateral views, optical sections from 3-dimensional datasets at the proximal-distal midpoint of the lens. Cyan dashed lines in A-P outline optic fissure margins. At 36 hpf and 48 hpf, *dzip1*^*-/-*^ embryos display wider optic fissure openings (F, G, N, O, *magenta arrowheads*). At 72 hpf, all mutants (9/9) still have an open fissure (P, *magenta arrowhead*), while 2/15 siblings have an open fissure. (Q-S) Quantification of optic fissure opening angle at 24 hpf, 36 hpf, and 48 hpf. n (embryos) for each genotype shown at the base of the graph. *P*-values were calculated using Welch’s t-test (Q-S). Scale bar: 50 μm. D, dorsal; V, ventral; N, nasal; T, temporal.

We sought to determine when a defect in the optic fissure first arises. We acquired 3-dimensional datasets of live wild type and *dzip1* mutant embryos at key stages in optic fissure development: 24, 36, 48, and 72 hpf (Fig 2A–2P). Lateral views of 3-dimensional renderings reveal whole-eye morphology (Fig 2A–2H), while cutaways to the lens center (Fig 2I–2P) provide a landmark position at which to measure the optic fissure opening angle, a metric of optic fissure formation and fusion.

We first visualized and quantified optic fissure opening angle at 24 hpf (Fig 2A, 2E, 2I, 2M and 2Q), to determine whether the first step, optic fissure formation, is affected. The optic fissure formed in both wild type and *dzip1* mutant embryos, with no significant difference in the optic fissure opening angle (Fig 2Q; wild type siblings 24.68±6.75°; *dzip1* mutants 20.57±6.47°). This suggests that the optic fissure initially forms normally in the absence of cilia and dzip1 function. At 36 hpf, after some eye growth and neurogenesis, in wild type embryos, the optic fissure margins were in close apposition at the ventral side of the eye (Fig 2B and 2J). In *dzip1* mutants, this was not observed: the optic fissure opening angle was significantly larger compared to wild type siblings (Fig 2F, 2N and 2R; wild type siblings 13.4±4.14°; *dzip1* mutants 29.82±7.15°). This defect persisted at 48 hpf (Fig 2C, 2G, 2K and 2O): *dzip1* mutants had a larger optic fissure opening compared to wild type siblings (Fig 2S; wild type siblings 3.44±1.18°; *dzip1* mutants 15.55±8.25°). We examined the optic fissure again at 72 hpf to determine whether there might simply be a delay in optic fissure closure (Fig 2D, 2H, 2L and 2P); at this stage, 9/9 (100%) *dzip1* mutants still exhibited an open optic fissure, compared to 2/15 (13.3%) sibling embryos. These data suggest that early stages of optic fissure formation appear to occur normally in *dzip1* mutant embryos, but optic fissure closure defects, as assayed by opening angle, are apparent by 36 hpf and persist beyond at least 72 hpf.

### Apoptotic cell death is not upregulated in dzip1 mutants

**Fig 3:**
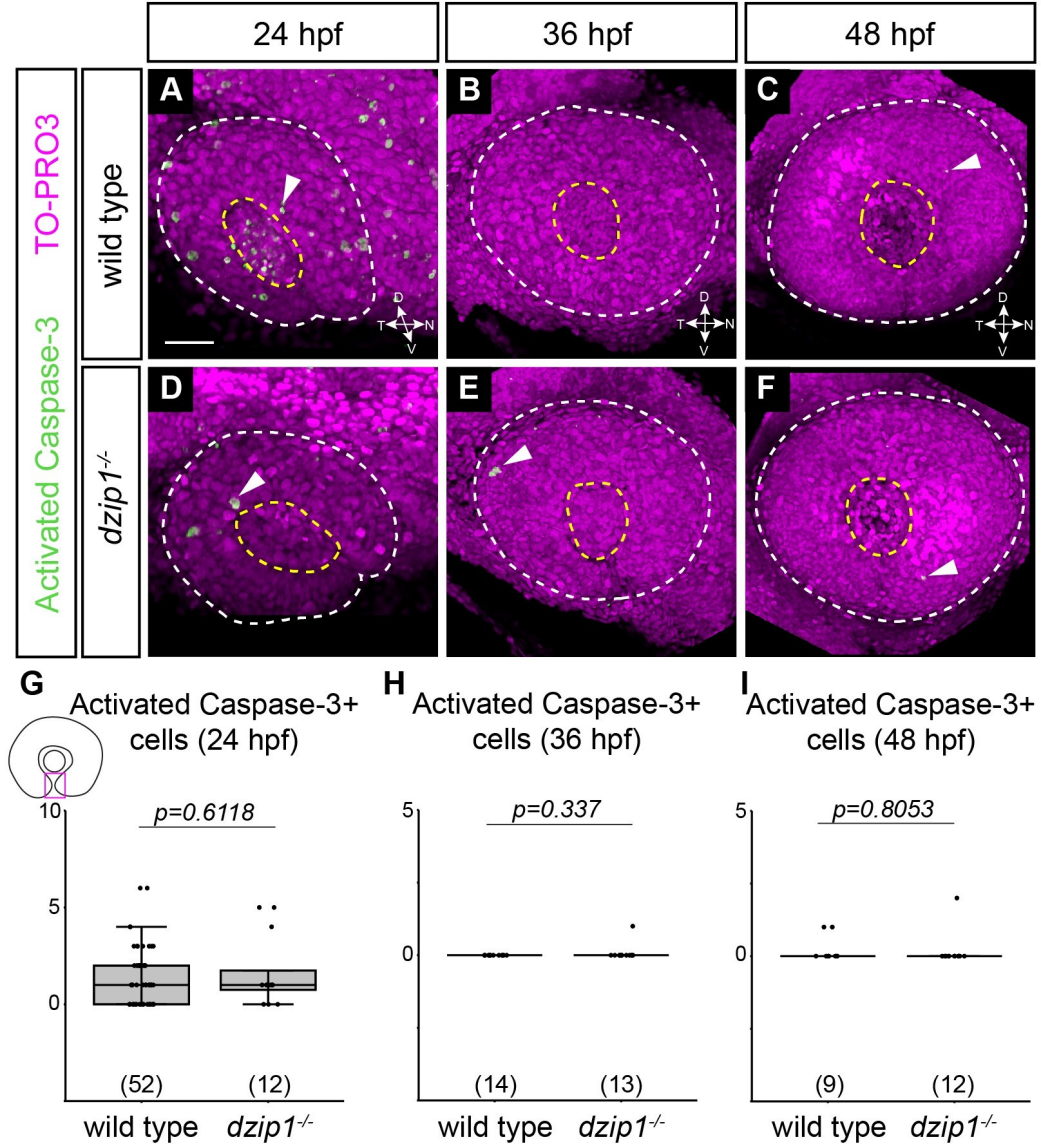
Apoptotic cell death in the optic fissure is not aberrantly upregulated in the *dzip1* mutant. (A-F) Whole mount immunofluorescence in wild type (A-C) and *dzip1*^*ts294e*^ mutants (D-F) for cell death (*green*; activated Caspase-3) and nuclei (*magenta*; TO-PRO-3) at 24 hpf (A, D), 36 hpf (B, E), and 48 hpf (C, F). All images are lateral views of 3-dimensional renderings. White dashed lines mark the boundary of the eye; yellow dashed lines mark the lens. Examples of cells labeled with activated caspase-3 elsewhere within the eye are indicated (*white arrowheads*). (G-I) Quantification of total number of activated caspase-3-positive cells within the optic fissure in the eye at 24 hpf, 36 hpf, and 48 hpf. n (embryos) for each genotype shown at the base of the graph. *P*-values were calculated using Welch’s t-test (M-O). Scale bar: 50 μm. D, dorsal; V, ventral; N, nasal; T, temporal.

To begin to determine the cellular basis of the coloboma defect in *dzip1* mutant embryos, we assayed apoptotic cell death. Aberrant cell death has been reported to be associated with coloboma in other genetic models [18], therefore, we sought to determine whether cell death is upregulated in the optic fissure of *dzip1* mutants. We assayed apoptotic cell death via antibody staining for the apoptotic marker activated caspase-3. Embryos were stained at 24, 36, and 48 hpf (Fig 3A–3F), and activated caspase-3-positive cells were manually counted through the depth of the optic fissure in the optic cup.

Although dying cells were observed sporadically throughout other parts of the developing eye and brain (Fig 3A–3F, *arrowheads*), there were very few apoptotic cells in the optic fissure at any timepoint assayed. Further, we found no significant difference in the number of apoptotic cells in the optic fissure between wild type siblings and *dzip1* mutants at 24 hpf (Fig 3G; wild type siblings, 1.37±1.52 cells; *dzip1* mutants, 1.67±1.87 cells), 36 hpf (Fig 3H; wild type siblings 0±0 cells; *dzip1* mutants 0.08±0.28 cells), or 48 hpf (Fig 3I; wild type siblings 0.22±0.44 cells; *dzip1* mutants 0.17±0.58 cells). These data suggest that aberrant, upregulated apoptotic cell death is unlikely to underlie the coloboma phenotype in *dzip1* mutants.

One limitation to this experiment is that this type of antibody staining only captures apoptotic cells at a specific timepoint, therefore, there could be alterations in cell death at timepoints that were not assayed. Despite this, the striking lack of apoptosis at any of the timepoints examined suggests that apoptotic cell death has little involvement in this phenotype.

### Cell proliferation is not altered in dzip1 mutants

**Fig 4:**
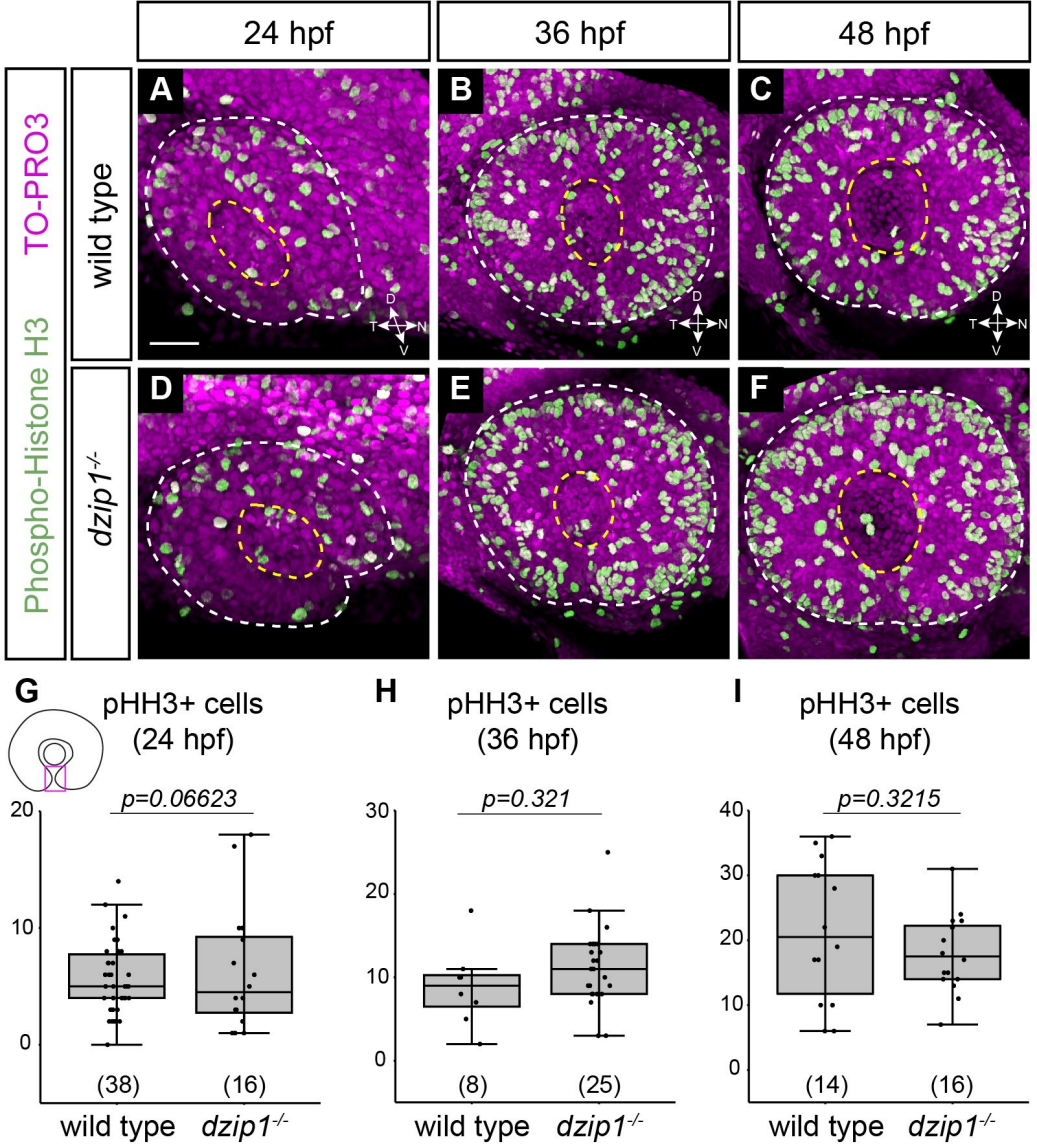
Cell proliferation in the optic fissure is not altered in the *dzip1* mutant. (A-F) Whole mount immunofluorescence in wild type (A-C) and *dzip1*^*ts294e*^ mutants (D-F) for cell death (*green*; phospho-histone H3) and nuclei (*magenta*; TO-PRO-3) at 24 hpf (A, D), 36 hpf (B, E), and 48 hpf (C, F). All images are lateral views of 3-dimensional renderings. White dashed lines mark the boundary of the eye; yellow dashed lines mark the lens. (G-I) Quantification of total number of phospho-histone H3-positive cells within the optic fissure in the eye at 24 hpf, 36 hpf, and 48 hpf. n (embryos) for each genotype shown at the base of the graph. *P*-values were calculated using Welch’s t-test (M-O). Scale bar: 50 μm. D, dorsal; V, ventral; N, nasal; T, temporal.

Another cellular mechanism that could potentially underlie the coloboma phenotype is altered proliferation, as the presence of too many or too few cells could disrupt the ability of the tissue to properly undergo fusion. We assayed proliferation via antibody staining for phospho-histone H3 (pHH3), which marks cells in G2/M. Embryos were stained at 24, 36, and 48 hpf (Fig 4A–4F), to determine whether alterations in cell proliferation might be apparent in *dzip1* mutants.

Cell proliferation was abundant throughout the eye and brain, and proliferating cells specifically in the optic fissure were also easily visualized. We manually counted mitotic (pHH3^+^) cells through the depth of the optic fissure in the optic cup and found no significant difference in the number of mitotic cells in the optic fissure between wild type siblings and *dzip1* mutants at 24 hpf (Fig 4G; wild type siblings 5.68±3.03 cells; *dzip1* mutants 6.31±5.31 cells), 36 hpf (Fig 4H; wild type siblings 8.88±4.73 cells; *dzip1* mutants 10.88±4.88 cells), or 48 hpf (Fig 4I; wild type siblings 21.36±10.75 cells; *dzip1* mutants 18.06±5.98 cells). These data suggest that alterations in cell proliferation are unlikely to underlie the coloboma phenotype in *dzip1* mutants.

### The optic fissure basal lamina fails to break down in dzip1 mutants

Optic fissure development proceeds via a series of conserved molecular and morphogenetic events [9–15]. Without an obvious change in cell death or proliferation, we turned our attention to determining which events in optic fissure development are impaired in *dzip1* mutants. During optic fissure closure, the basal lamina or basement membrane that lines the tissue margins must break down in order for the two sides of the fissure to come into direct contact and allow for cell rearrangements and tissue fusion.

**Fig 5:**
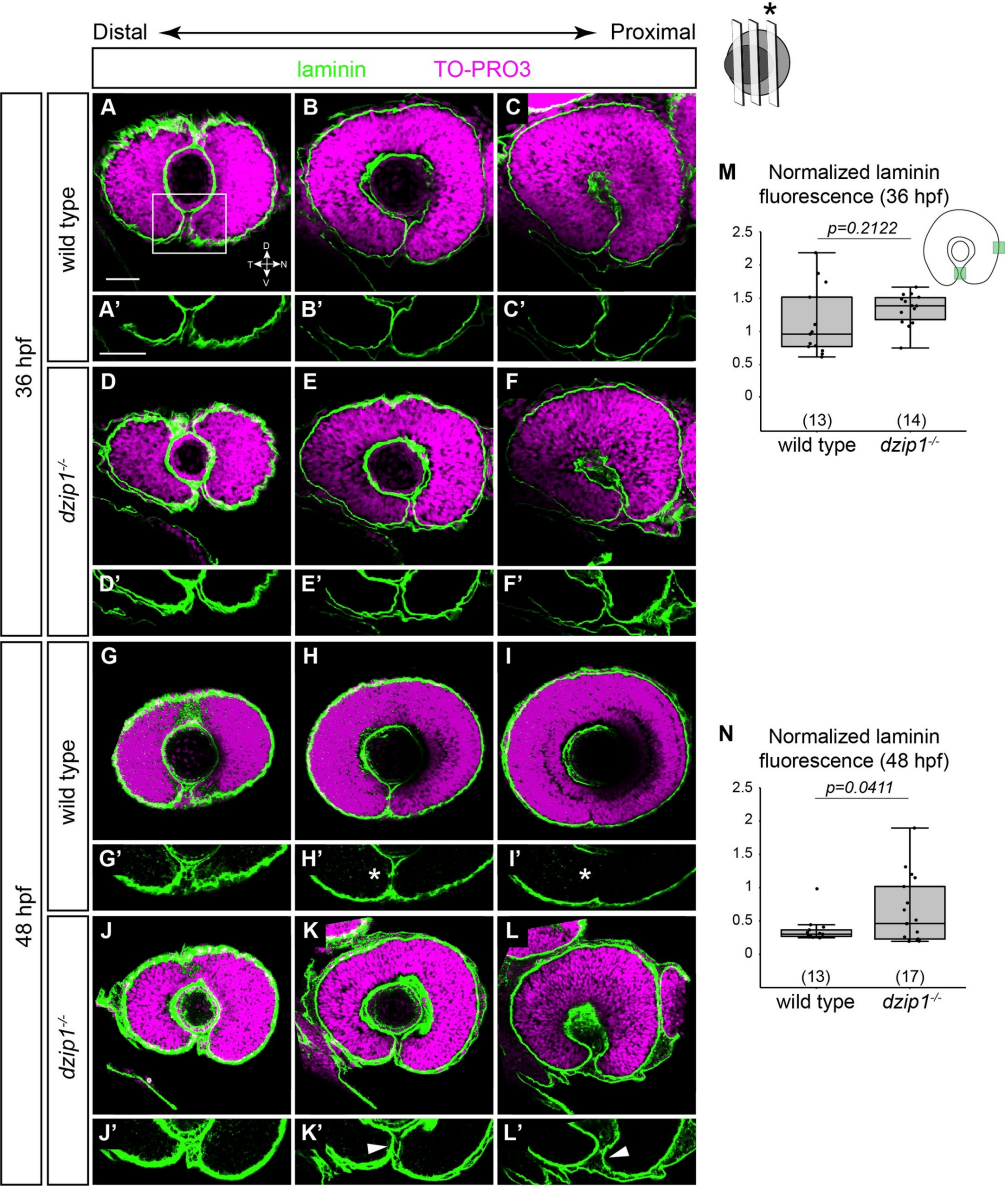
The optic fissure basement membrane fails to breakdown in *dzip1* mutants. (A-L) Whole mount immunofluorescence for Laminin (*green*), and nuclei (*magenta*, TO-PRO-3) in wild type (A-C, G-I) and *dzip1*^*ts294e*^ mutants (D-F, J-L) at 36 hpf (A-F), and 48 hpf (G-L). All images are lateral views. Single optical sections were obtained from three different depths along the proximal-distal axis of the eye (see Methods for details). (A’-L’) Insets of the optic fissure, the laminin channel alone (*green*) for wild type (A’-C’, G’-I’), and *dzip1*^*ts294e*^ mutants (D’-F’, J’-L’), at 36 hpf (A’-F’) and 48 hpf (G’-I’). At 36 hpf, laminin protein completely lines the fissure in both genotypes. At 48 hpf, laminin protein is lost in the optic fissure in wild type embryos (H’, I’, *white asterisks*), whereas laminin continues to persist in the optic fissure in *dzip1*^*-/-*^ embryos (K’, L’, *white arrowheads*). (M, N) Quantification of laminin fluorescence intensity in the optic fissure in the proximal optical section at 36 hpf and 48 hpf. Laminin fluorescence intensity at the optic fissure was normalized to the laminin fluorescence intensity at the nasal boundary of the eye next to the olfactory placode. n (embryos) for each genotype shown at the base of the graph. *P*-values were calculated using Welch’s t-test (M-O). Scale bar: 50 μm. D, dorsal; V, ventral; N, nasal; T, temporal.

To begin to determine what step of optic fissure development is disrupted in *dzip1* mutants, we assayed basal lamina breakdown via antibody staining for the core extracellular matrix component, laminin. Embryos were analyzed at 36 and 48 hpf (Fig 5A–5L): at 36 hpf in wild type embryos, basement membrane breakdown is initiated at specific locations in the optic fissure, whereas by 48 hpf, significant basement membrane breakdown has occurred. 3-dimensional imaging data were acquired to allow for visualization and quantitative analysis of the basal lamina at different depths of the optic fissure.

At 36 hpf, in wild type sibling embryos (Fig 5A–5C’), laminin was visualized lining the optic fissure margins at distal, medial, and proximal sections through the optic cup. In *dzip1* mutants (Fig 5D–5F’), laminin also lined both margins of the optic fissure. Laminin fluorescence intensity was quantified in the proximal section behind the lens (Fig 5B and 5E) of wild type sibling and *dzip1* mutant embryos. At 36 hpf, in the proximal section behind the lens, we found no significant difference in laminin fluorescence intensity between wild type and mutants (Fig 5M; wild type siblings 1.33±0.24; *dzip1* mutants 1.13±0.52). At 48 hpf, in the wild type optic fissure, laminin fluorescence appeared discontinuous, and even absent in the proximal section, a sign that basement membrane breakdown had occurred (Fig 5G–5I, asterisks). In contrast, laminin still appeared to completely line the optic fissure margins in *dzip1* mutants (Fig 5J–5L, arrowheads). We quantified these results at the proximal position (Fig 5N): laminin fluorescence intensity was significantly higher in the *dzip1* mutants compared to wild type siblings (wild type siblings 0.36±0.2; *dzip1* mutants 0.65±0.5). These results indicate that in *dzip1* mutants, basement membrane breakdown does not occur appropriately.

### Neural crest cells do not properly populate the optic fissure in dzip1 mutants

We next considered potential underlying causes of impaired basement membrane breakdown and disrupted optic fissure closure. The periocular mesenchyme (POM) is a heterogeneous extraocular migratory cell population comprised of neural crest and also mesodermally-derived migratory cells [63, 64]. The POM has been implicated in optic fissure closure [15, 65–68]: electron microscopy revealed POM cells near sites where basement membrane breakdown were taking place and transplanting the eye to sites distant from POM led to failure of optic fissure closure [11, 14, 69]. Further, recent work has proposed that POM may be a source of matrix metalloproteinases to degrade basement membrane [70]. Therefore, disruption to POM localization could be a cause of optic fissure closure defects, and therefore coloboma, in the *dzip1* mutant.

**Fig 6:**
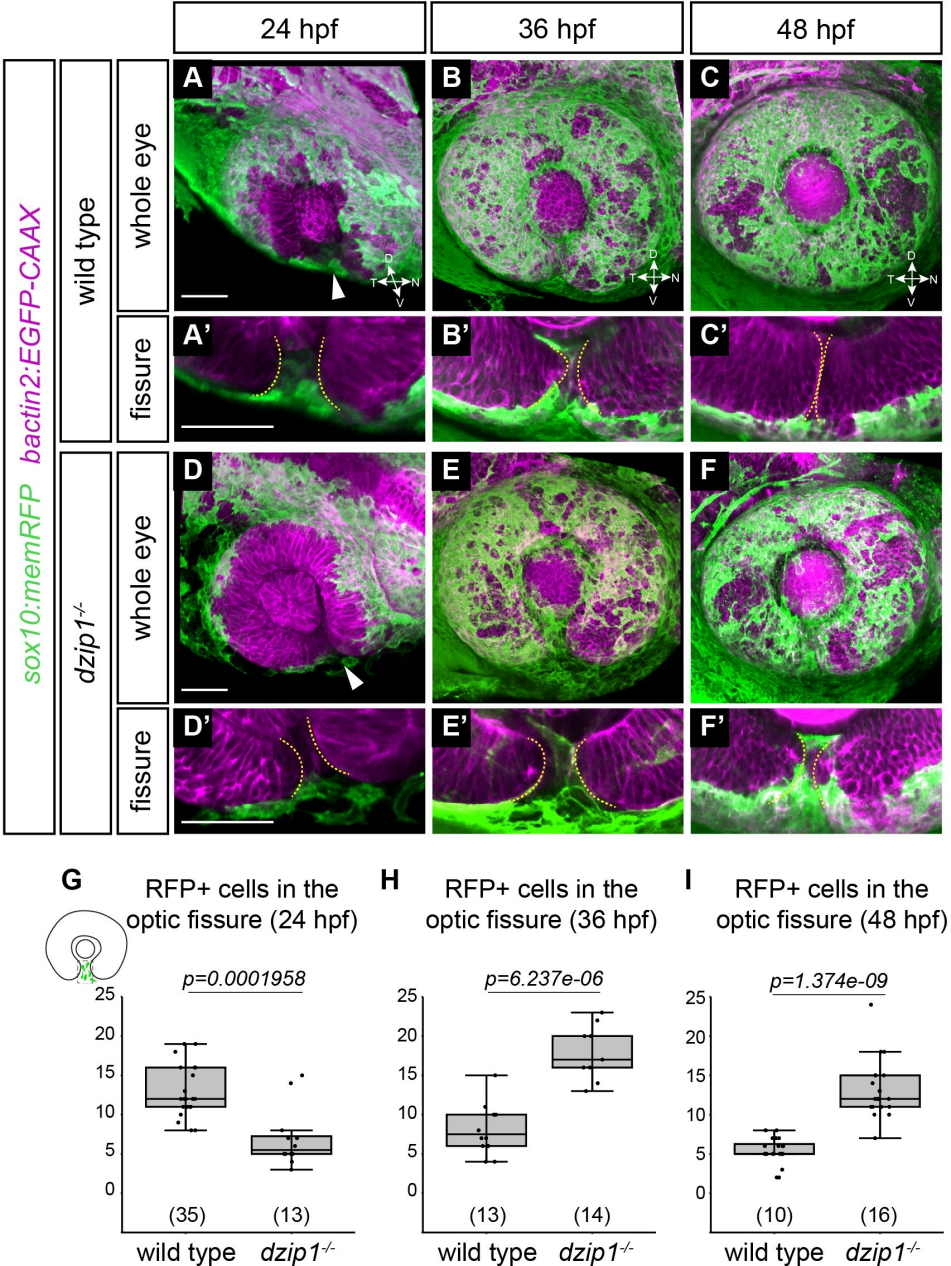
*Tg(sox10*:*memRFP)-*positive neural crest cells do not properly localize in the optic fissure in *dzip1* mutants. Embryos visualized for neural crest (*green*; *Tg(sox10*:*memRFP)*) and cell membranes (*magenta*; *Tg(bactin2*:*EGFP-CAAX)*) in wild type (A-C) and *dzip1*^*ts294e*^ mutants (D-F) at 24 hpf (A, D), 36 hpf (B, E), and 48 hpf (C, F). (A’-F’) Zoomed views of images in A-F to facilitate visualization of neural crest cells within the optic fissure. All images are lateral views of 3-dimensional renderings. Some *Tg(sox10*:*memRFP)*-positive neural crest cells are detectable in the optic fissure by 24 hpf in both wild type and *dzip1*^*-/-*^ embryos (*white arrowheads* in A, D). (G-I) Quantification of total number of *Tg(sox10*:*memRFP)*-positive cells through the length of the optic fissure within the eye at 24, 36, and 48 hpf. n (embryos) for each genotype shown at the base of the graph. *P*-values were calculated using Welch’s t-test (M-O). Scale bar: 50 μm. D, dorsal; V, ventral; N, nasal; T, temporal.

We evaluated POM recruitment to the optic fissure region using two different transgenes. The first was *Tg(sox10*:*memRFP)*, which specifically labels neural crest cells. At 24, 36, and 48 hpf, we acquired 3-dimensional z-stacks of the eye of both wild type siblings and *dzip1* mutants (Fig 6A–6F). Zoomed views (Fig 6A’–6F’) show neural crest cells in the fissure. We manually counted the number of neural crest cells (*sox10* transgene-positive) specifically in the optic fissure through the depth of the optic cup (Fig 6G–6I).

In wild type siblings, neural crest cells were recruited to the optic fissure by 24 hpf (Fig 6A, 6A’ and 6G), and then decreased in number as the optic fissure closed (Fig 6H and 6I). In *dzip1* mutants, at 24 hpf, significantly fewer *sox10* transgene-positive cells were in the optic fissure (Fig 6D, 6D’ and 6G; wild type siblings, 12.9±3.35 cells; *dzip1* mutants 7±3.77 cells), suggesting that the initial recruitment of neural crest cells was defective when cilia were absent. By 36 and 48 hpf, however, *dzip1* mutants appeared to have significantly more *sox10* transgene-positive cells in the optic fissure compared to their wild type sibling counterparts (Fig 6H and 6I; at 36 hpf: wild type siblings 8.17±3.19 cells; *dzip1* mutants 17.89±3.52 cells; at 48 hpf: wild type siblings 5.5±1.62 cells; *dzip1* mutants 13.14±3.6 cells). This suggests that although initial localization of neural crest cells to the optic fissure is defective, the cells can accumulate there, albeit in a delayed manner. Their retention, in turn, may be a secondary effect of the failure of the optic fissure to close, as neural crest cells may exit the fissure as it closes. Overall, this result suggests that in *dzip1* mutants, an initial defect may be the failure of neural crest cells to properly populate the optic fissure as it forms.

### Endothelial POM cells aberrantly accumulate in the optic fissure in dzip1 mutants

Having evaluated the neural crest subpopulation of the POM, and especially having uncovered a defect in neural crest recruitment to the optic fissure, we sought to assess a distinct subpopulation of the POM, endothelial cells. To do this, we took advantage of the *Tg(kdrl*:*mCherry-ras)* transgenic line, which marks endothelial cells. Endothelial cells first migrate around the dorsal and nasal aspect of the optic cup and enter into the optic fissure and the space behind the lens around 24 hpf. There, the endothelial cells form the hyaloid vasculature, with the hyaloid vessel in the optic fissure. By 36 and 48 hpf, the optic fissure fuses around the hyaloid vessel; in addition, the superficial ocular vasculature becomes more elaborated.

**Fig 7:**
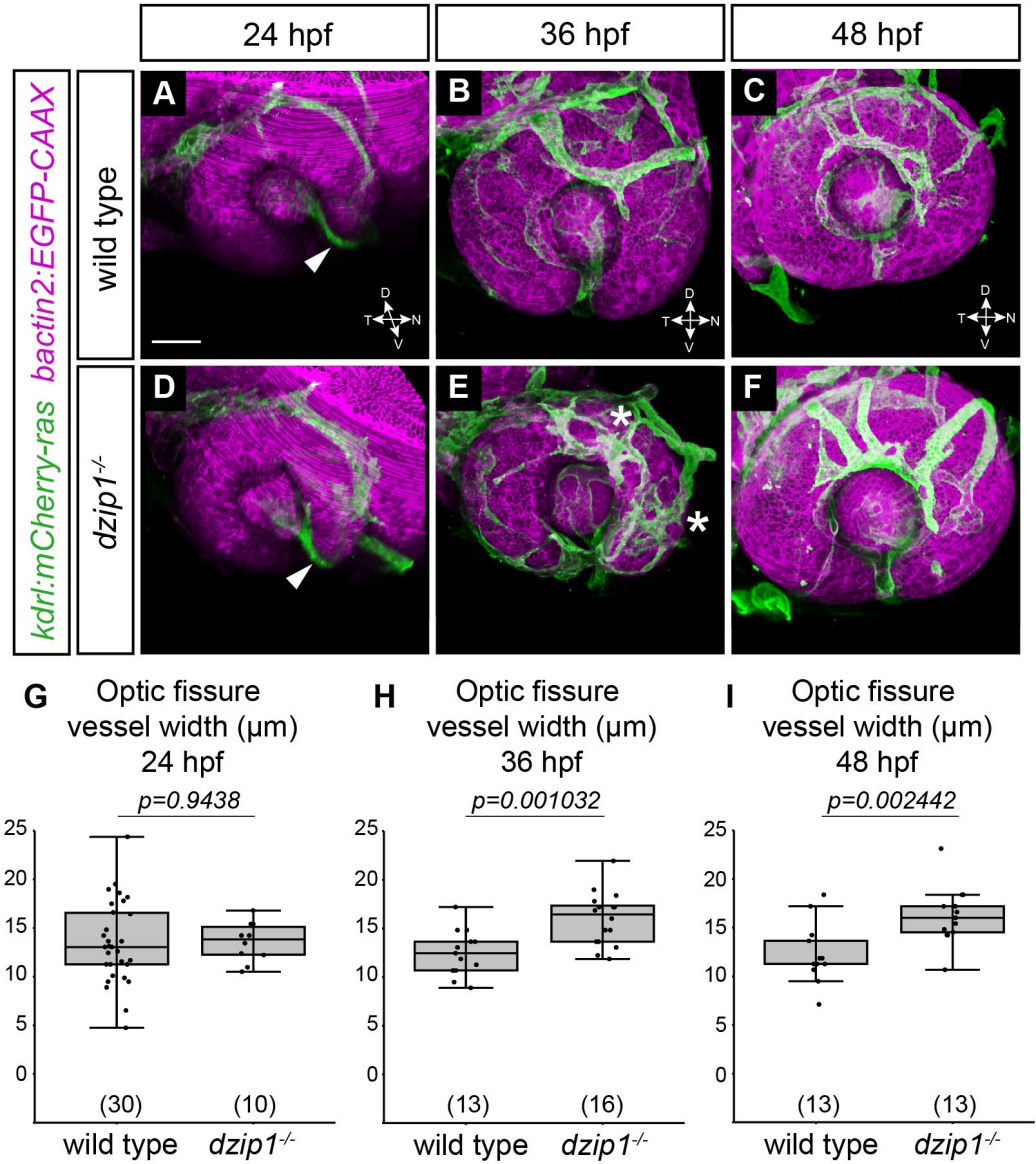
*dzip1* mutants exhibit defective accumulation of POM-derived endothelial cells. (A-F) Embryos visualized for endothelial cells (*green*; *Tg(kdrl*:*ras-mCherry)*) and cell membranes (*magenta*; *Tg(bactin2*:*EGFP-CAAX)*) in wild type (A-C) and *dzip1*^*ts294e*^ mutants (D-F) at 24 hpf (A, D), 36 hpf (B, E), and 48 hpf (C, F). All images are lateral views of 3-dimensional renderings. mCherry-positive endothelial cells populate the optic fissure by 24 hpf in both wild type and *dzip1*^*-/-*^ embryos (A, D; *white arrowheads*). (E) *asterisks*, ectopic branching of the superficial network in *dzip1* mutant. (G-I) Quantification of fissure vessel width (in μm) at 24 hpf, 36 hpf, and 48 hpf. n (embryos) for each genotype shown at the base of the graph. *P*-values were calculated using Welch’s t-test (M-O). Scale bar: 50 μm. D, dorsal; V, ventral; N, nasal; T, temporal.

By crossing the *Tg(kdrl*:*mCherry-ras)* into the *dzip1* mutant background, we evaluated the development of the ocular vasculature in wild type siblings and *dzip1* mutants. We acquired 3-dimensional datasets of both wild type siblings and *dzip1* mutants at 24, 36, and 48 hpf (Fig 7A–7F). At 24 hpf, endothelial cells were found in the optic fissure and the space behind the lens in both wild type siblings and *dzip1* mutants (Fig 7A and 7D). We quantified these data by measuring the widest width of the hyaloid vessel. At 24 hpf, there was no difference in hyaloid vessel width between wild type siblings and *dzip1* mutants (Fig 7G; wild type siblings 13.49±4.22 μm; *dzip1* mutants 13.56±2.04 μm), suggesting that initial formation of the hyaloid vessel is unaffected in *dzip1* mutants. At 36 hpf, the hyaloid vasculature continued to develop, and the superficial ocular vasculature network was elaborated (Fig 7B and 7C). The hyaloid vessel was present in both wild type siblings and *dzip1* mutants, although upon quantification, the hyaloid vessel was wider in mutants compared to wild type siblings (Fig 7H; wild type siblings 12.5±2.36 μm; *dzip1* mutants 15.98±2.3 μm). At 48 hpf, the hyaloid vessel defect persisted (Fig 7E and 7F), and quantification demonstrated that the hyaloid vessel remained wider in mutants compared to wild type siblings (Fig 7I; wild type siblings 12.27±3.01 μm; *dzip1* mutants 16.04±2.93 μm). Separate from the optic fissure, in some *dzip1* mutants, there appeared to be some aberrations in the superficial ocular vasculature, including some ectopic branches compared to wild type siblings (Fig 7B and 7E, *asterisks*). Specifically, at 48 hpf, wild type embryos typically have two (occasionally three) dorsal branches: in our datasets, 10/13 siblings had two dorsal branches, while 3/13 had three. In contrast, all 13/13 dzip1 mutants had at least three abnormal dorsal branches with aberrant morphology. Similar to the later phenotype observed with neural crest cell recruitment, it is possible that the wider hyaloid vessel width might be a secondary effect of the failure of optic fissure closure.

Taken together, these results suggest that initial recruitment of endothelial cells to the optic fissure is unaffected in the *dzip1* mutant, although later in optic fissure development, the hyaloid vessel is wider than that of wild type sibling embryos.

### Loss of cilia elicits variable effects on Hh signaling in the eye

In many tissues, cilia are crucial subcellular structures in which key Hedgehog (Hh) signaling events occur [21–25]. Both alterations in Hh signaling and disruptions to cilia can be associated with coloboma [30–33, 56, 71], however it remains unknown if these are directly related. In the *dzip1* mutant, loss of cilia has previously been demonstrated to lead to a loss of Hh signaling [45–47], although somewhat surprisingly, a role for cilia in Hh signaling in the early eye has not been directly examined; additionally, dzip1 can function in Hh signaling independent of cilia, by regulating Gli turnover. We therefore sought to determine whether the *dzip1* mutant might alter Hh signaling in the eye.

**Fig 8:**
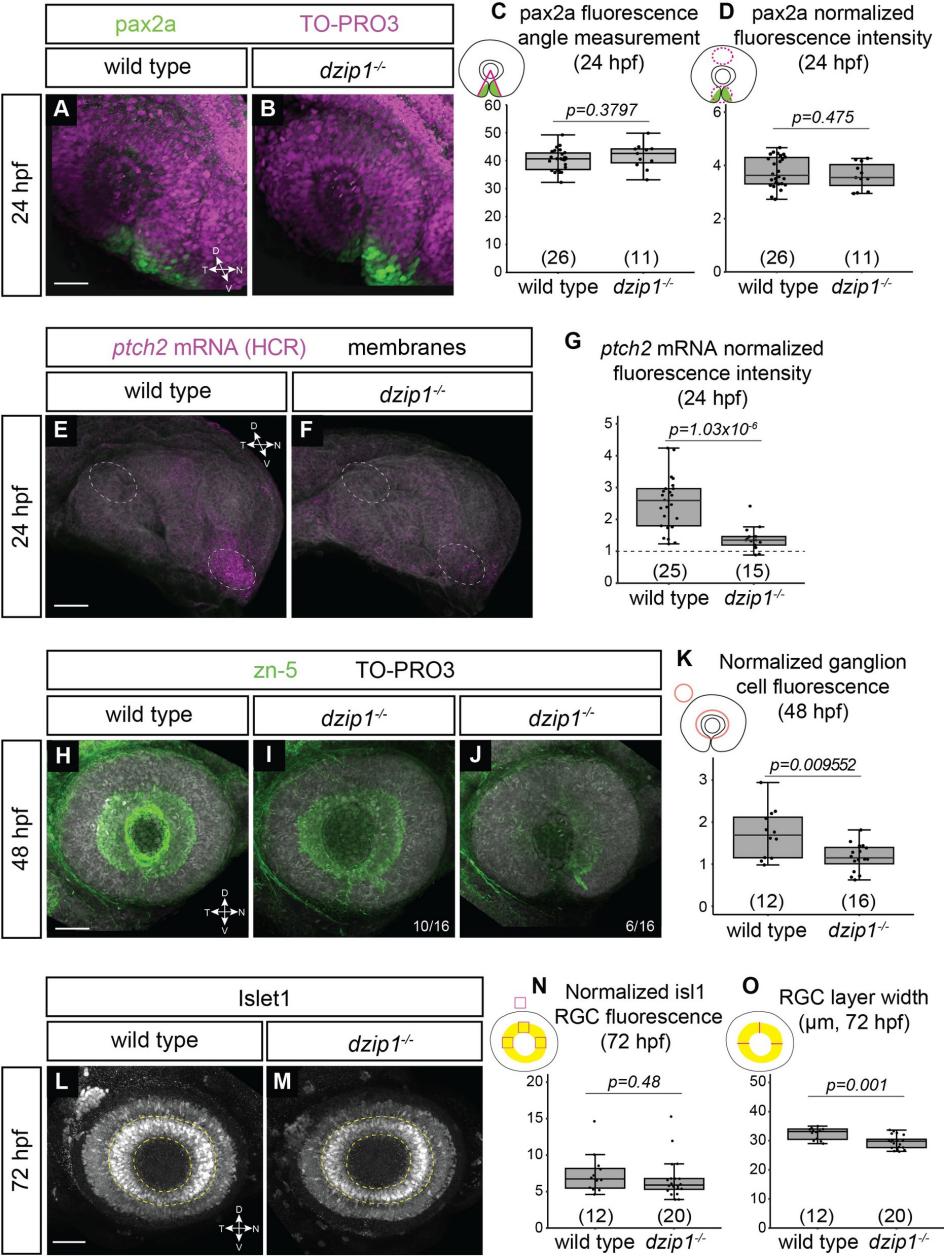
*dzip1* mutants show defects in ganglion cell differentiation, which may be indicative of disrupted Hedgehog (Hh) signaling. (A-B) Whole mount immunofluorescence for pax2a (*green*), and nuclei (*magenta*, TO-PRO-3) in wild type (A) and *dzip1*^*ts294e*^ mutants (B) at 24hpf. Images are lateral views of 3-dimensional renderings. (C) Quantification of the pax2a+ domain, and (D) Quantification of pax2a normalized fluorescence intensity at 24 hpf. Quantification was carried out as depicted in the schematics; for fluorescence quantification, measurements in the optic stalk and fissure were normalized to a region in the dorsal optic cup. (E-F) *ptch2* mRNA expression (HCR, *magenta*) and membranes (*gray*) in wild type (E) and *dzip1*^*ts294e*^ mutants (F) at 24 hpf. Images are maximum intensity projections of *ptch2* mRNA channel (magenta) on an average projection of the membrane channel (gray) to visualize tissue morphology; embryos were oriented laterally. (G) Quantification of *ptch2* mRNA fluorescence intensity. Fluorescence in the optic stalk region was normalized to a region in the dorsal optic cup (as depicted by dashed ellipses in E and F). (H-J) Whole mount immunofluorescence for retinal ganglion cells (*green*, zn-5) and nuclei (*magenta*, TO-PRO-3) in wild type (H) and different *dzip1*^*ts294e*^ mutants (I, J) at 48 hpf. All images are lateral views of 3-dimensional renderings. Two classes of *dzip1*^*-/-*^ phenotype were noted (I, J); fraction of *dzip1*^*-/-*^ embryos showing each type of zn-5 labeling is shown at the bottom right of each panel. (K) Quantification of zn-5 fluorescence intensity at 48 hpf. Fluorescence intensity was measured in an elliptical ROI on a maximum intensity projection of the three-dimensional z-stack spanning the depth of the lens. Zn-5 fluorescence intensity in the eye was normalized to a region in the brain dorsal to the eye. (L-M) Whole mount immunofluorescence for islet1/2 (*gray*) in wild type (L) and *dzip1*^*ts294e*^ mutants (M) at 72 hpf. Images are maximum intensity projections of laterally oriented embryos. Islet1 is expressed by retinal ganglion cells (layer marked by yellow dashed lines) and subsets of amacrine, bipolar, and horizontal cells. (N-O) Quantification of islet1/2 staining in RGCs, specifically for fluorescence intensity (N) and RGC layer width (O). Quantification method is depicted in each schematic. For islet1/2 RGC fluorescence, mean gray value was measured at nasal, dorsal, and temporal positions within the RGC layer, and the average of those values was normalized to the mean gray value at a position dorsal to (outside of) the eye. The width of the RGC layer was calculated by measuring the width of the RGC layer at nasal, dorsal, and temporal positions; these measurements were averaged for each embryo. For all graphs, n (embryos) for each genotype shown at the base of the graph. *P*-values were calculated using a student’s t-test (M-O). Scale bar: 50 μm. D, dorsal; V, ventral; N, nasal; T, temporal.

To determine whether Hh signaling is altered in the *dzip1* mutant during early eye development, we carried out antibody staining at 24 hpf for the Hh target gene pax2a, which is expressed in the ventral eye and optic stalk. Alterations in Hh signaling commonly perturb the size of the domain and the level to which pax2a is expressed [56, 71–73]. Somewhat surprisingly, we observed no difference in the size of the optic cup domain in which pax2a is expressed (Fig 8A and 8B), and we quantified this result by measuring the sector of the optic cup (as an angle measurement) in which pax2a staining is present (Fig 8C; wild type siblings 40.26±3.77°; *dzip1* mutants 41.67±4.56°). We also asked whether the level of pax2a expression was affected by loss of *dzip1*.We measured the fluorescence intensity of pax2a staining in the optic fissure and stalk region (normalized to fluorescence in a control region, the dorsal eye) and found no difference in the relative fluorescence intensity of pax2a staining in wild type compared to *dzip1* mutants (Fig 8D; wild type siblings 3.76±0.57; *dzip1* mutants 3.61±0.5). These results suggest that pax2a expression is not affected by the loss of *dzip1*.

Pax2a is only one target of Hh signaling in the early eye, therefore, we sought to examine another target, *ptch2*. We visualized *ptch2* mRNA expression using HCR fluorescence in situ hybridization in wild type and *dzip1* mutant embryos (Fig 8E and 8F). We measured the fluorescence intensity of ptch2 HCR signal and normalized the signal in the optic fissure and stalk region to a control region without *ptch2* expression, the dorsal eye (ellipses in Fig 8E and 8F). In contrast to pax2a, relative fluorescence intensity of *ptch2* expression is significantly decreased in *dzip1* mutants compared to wild type siblings (Fig 8G; wild type 2.5±0.82; dzip1 mutants 1.38±0.37), suggesting that Hh signaling is impaired. These data indicate that different Hh target genes during early eye development may be affected differently by loss of *dzip1*.

Hh signaling is also involved in retinal neurogenesis [74–78], during which a wave of Shh expression precedes retinal ganglion cell differentiation. Therefore, examining RGC differentiation could provide another means to evaluate perturbations of Hh signaling. We visualized RGC differentiation using staining with the antibody zn-5, which recognizes the cell adhesion molecule alcama and labels retinal ganglion cell bodies, dendrites, and axons [43, 79]. At 48 hpf in wild type embryos, an array of zn-5 labeled RGCs was apparent (Fig 8H). In *dzip1* mutants, however, we saw variable staining: in some embryos (10/16), an RGC array was present, although with diminished fluorescence; in other embryos (6/16), the RGC array itself was diminished, with staining most apparent only in the ventronasal quadrant of the optic cup, where RGC differentiation commences (Fig 8I and 8J). This variability could be similar to the incomplete penetrance observed in the coloboma phenotype. We quantified the RGC fluorescence and found that indeed, zn-5 staining is diminished in *dzip1* mutants compared to wild type siblings (Fig 8K; normalized RGC fluorescence in wild type siblings 1.72±0.59; *dzip1* mutants 1.17±0.33).

We were curious as to whether such a defect in RGC differentiation might persist, so we sought to examine a second marker of RGC development at a different timepoint. Islet1 is expressed by RGCs as well as subsets of differentiated amacrine, bipolar, and horizontal cells. We performed immunofluorescence using an antibody that recognizes Islet1/2 in wild type sibling and *dzip1* mutant embryos at 72 hpf and identified the RGC layer based on its position in the retina (Fig 8L and 8M; RGC layer marked with yellow dashed lines). Islet1/2 staining was quantified in two ways: first, as schematized in Fig 8N, we measured Islet1/2 RGC fluorescence intensity by averaging the fluorescence intensity at three positions in the RGC layer (nasal, temporal, and dorsal), and normalizing this to the fluorescence intensity at a control position dorsal to (outside of) the eye. These measurements revealed that relative Islet1/2 fluorescence intensity was not significantly changed between *dzip1* mutants and wild type siblings (Fig 8N; wild type 7.42±2.75; *dzip1* mutants 6.7±2.75). In addition to Islet1/2 fluorescence intensity, we also measured the thickness of the RGC layer as marked by Islet1/2: as schematized in Fig 8O, the width of the RGC layer was measured at three positions (nasal, temporal, and dorsal), and these measurements were averaged for each embryo. As marked by Islet1/2, the RGC layer was slightly but significantly thinner in *dzip1* mutants compared to sibling controls (Fig 8O; wild type 32.42±2.04 μm; *dzip1* mutants 29.55±2.32 μm). We interpret these results to suggest that Islet1/2 is expressed to the same level in both wild type siblings and *dzip1* mutants, however, fewer RGCs differentiate in dzip1 mutants, resulting in a thinner RGC layer.

Taken together, these results suggest that loss of *dzip1* causes variable effects on Hh target gene expression in early eye development, but RGC differentiation, another Hh-dependent process, is impaired.

## Discussion

Cilia are crucial for development and proper physiology in a wide range of tissues. In the eye, the cilia machinery is required for vision, particularly in the context of photoreceptor development and function: conserved components are an integral part of the connecting cilium, which links the inner and outer segments [26–28]. There remains a gap in our understanding of cilia function in other aspects of eye development, for example, early eye development has not been examined in zebrafish, as many cilia gene mutants retain cilia during early eye developmental stages due to maternal deposition of proteins and mRNAs.

Here, we present experimental data testing the role of *dzip1* in early eye development. Dzip1 is necessary for cilia formation and has cilia-independent roles in Hh signaling and centrosomes [50–53]. Notably, the zebrafish *dzip1* mutant exhibits a loss of cilia by 10 hpf [49], a time when the zebrafish eye just commences evagination, making this a useful system to ask questions regarding cilia and Hh signaling in optic cup morphogenesis, to understand how coloboma might arise in ciliopathy conditions, and how Hh signaling is affected in the zebrafish eye in the absence of cilia. We first confirmed the early loss of cilia in the developing eye (Fig 1A–1H), a tissue which had previously been unexplored in this mutant. We found that in the absence of *dzip1*, the optic cup forms correctly (Fig 2A and 2E), however, the embryos go on to exhibit coloboma, a structural defect due to disrupted development of the optic fissure (Fig 1I–1K). These observations indicate a defect in optic fissure closure: in *dzip1* mutants, the optic fissure margins fail to be closely apposed as the eye grows and neurogenesis proceeds, and the basement membrane fails to break down compared to wild type siblings (Figs 2 and 5). Cell proliferation and cell death were not changed in *dzip1* mutant optic fissures compared to wild type siblings (Figs 3 and 4), suggesting that alterations to these processes do not underlie the coloboma phenotype. In contrast, the neural crest, an extraocular cell population implicated in optic fissure basement membrane breakdown and closure [15, 65–68, 70], is not appropriately recruited to the optic fissure in *dzip1* mutants (Fig 6). Even though neural crest cells do not arrive at the appropriate time, at later timepoints, they do accumulate to a greater extent in *dzip1* mutants compared to wild type siblings, a finding observed in at least one other coloboma model with an optic fissure closure defect [80]. This suggests that even once the cells arrive at the optic fissure, they may not be able to execute their appropriate function in basement membrane breakdown. There may be multiple defects in this cell population, or failure of some aspect of crosstalk between neural crest cells and the optic fissure. Taken together, these results suggest that loss of *dzip1*, through its functions in primary cilia, Hedgehog signaling, or centrosomes, results in coloboma, and we speculate that an underlying cause may be the failure of neural crest cells to initially populate the optic fissure correctly.

How might loss of cilia lead to coloboma in the *dzip1* mutant? In mouse, dramatic eye morphogenesis defects have been described in the *arl13b* knockout [34, 35]. Many of these defects can be ascribed to defective Hh signaling, and a recent study indicated that arl13b can function in Hh signaling outside of cilia [36]. This is similar to the situation with dzip1, which is necessary for cilia formation, but also functions in Hh signaling outside of cilia [50–53]. To our surprise, a Hh-dependent transcriptional target, *pax2*, was induced normally in *dzip1* mutants during optic cup morphogenesis (Fig 8). Because loss of Hh signaling, as in the *smoothened* mutant, dramatically diminishes pax2a expression [72, 73], this result suggests that Hh signaling is still occurring in *dzip1* mutants. In contrast, induction of a different Hh target gene, *ptch2*, was severely diminished in *dzip1* mutants (Fig 8), suggesting the Hh signaling is impaired. Why would different Hh target genes be so differentially affected by loss of *dzip1*? One possibility is that loss of *dzip1* results in diminished, but not completely impaired Hh signaling, and this diminished signaling is still capable of inducing certain target genes such as pax2a. Transmission electron microscopy has revealed that the basal body still docks to the apical membrane in the zebrafish *dzip1* mutant [49]; therefore, it is possible that Hh signaling components, including arl13b, are capable of functioning in early eye development with an intact basal body despite the absence of a ciliary axoneme. An alternative possibility is that another gene, perhaps *dzip1l*, can partially compensate for loss of *dzip1*, allowing for expression of some target genes. Yet another alternative possibility is that despite prior data indicating the dependence of pax2a expression on Hh signaling, there may be an independent pathway that can compensate. Further work will be necessary to test these models.

In contrast to the early eye development observations, induction of retinal ganglion cells, another Hh-dependent process, was impaired in *dzip1* mutants, as assayed by zn-5 and Islet1/2 staining (Fig 8). These results are consistent with the idea that Hh signaling may have varying tissue-specific requirements for cilia at different times in development. This has been previously reported in zebrafish mutants for intraflagellar transport proteins *ift57*, *ift88*, and *ift172* [81], and it has also been shown that *dzip1* mutants exhibit gene expression changes that are only partially overlapping with cyclopamine treatment [82]. These observations merit further investigation into spatiotemporally regulated interactions of cilia and Hh signaling, as well as other functions for dzip1 or of cilia.

Disruptions to Hh signaling are known to be associated with coloboma. For example, the *ptch2* mutant, in which Hh signaling is overactive, exhibits coloboma [56, 71]. Upon discovering a coloboma phenotype in the *dzip1* mutants, we hypothesized that this might arise in a manner similar to the *ptch2* mutant. This appears not to be the case: in the *ptch2* mutant, cell movements responsible for initial stages of optic fissure and stalk formation are disrupted [56]; in the *dzip1* mutant, optic fissure and stalk formation appear normal. In addition, in the *ptch2* mutant, pax2a and *ptch2* expression are expanded and increased during optic cup morphogenesis stages [56, 71], while in the *dzip1* mutant, pax2a expression appears unaffected. Our evaluation of early eye development in the *dzip1* mutant suggests that patterning and morphogenesis of the optic cup proceed normally. This itself is somewhat surprising, demonstrating that dzip1’s functions in Hh signaling, cilia, and centrosomes may not be essential for early eye development. Even though we detected a dramatic decrease in *ptch2* expression in *dzip1* mutants (Fig 8), this did not disrupt eye formation. Further work will be necessary in order to dissect the molecular and cellular phenotypes underlying the *dzip1* mutant phenotype.

It is important to note that we have examined a constitutive *dzip1* mutant, in which all cells have lost cilia. Therefore, we do not know if the defect in neural crest cell recruitment is due to defects in the eye tissues, or in the neural crest cells themselves, or both. It is known that neural crest cells have primary cilia, which are necessary for proper development and differentiation. Neural crest mutants exhibit coloboma [83, 84], and neural crest cells are implicated in optic fissure closure [11, 15, 65–68, 70]. In mouse, loss of neural crest cilia leads to defective anterior segment development in the eye [85], and defects in ventral forebrain morphogenesis [86]. In both cases, Hh signaling in the neural crest cells is lost, and this is thought, at least in part, to contribute to the phenotype: for example, the ventral forebrain phenotype is reproduced via neural crest-specific deletion of *Smo*. In addition to the loss of Hh signaling, other cellular processes may be affected in neural crest in which cilia have been lost, including cell proliferation and migration, although this may vary between species [87, 88]. Loss of *dzip1* in neural crest cells may affect cilia, Hedgehog signaling, and centrosomes, all of which may play crucial roles in differentiation and directed migration to the correct target tissues. Future experiments, for example, cell transplantation in zebrafish, would be necessary to determine the tissues in which *dzip1* is required, and to determine which function (or functions) of dzip1 are crucial for optic fissure development.

Ciliopathies have revealed much about the developmental and physiological processes requiring cilia, and these varied conditions may be the key to revealing nuances underlying the biology. Coloboma is not usually associated with the most commonly studied ciliopathies, yet it is associated with Joubert/COACH syndrome and some oral-facial-digital syndromes [30–33, 89, 90]. It has not been clear whether these coloboma phenotypes are arising due to defects in eye tissues or neural crest (or both), and whether this is due to disrupted Hh signaling or other requirements for *dzip1*, for example, in proliferation or migration. Notably, the *MZift88* mutant, a different zebrafish mutant in which cilia were lost from early stages, did not appear to exhibit coloboma, although the eye was not examined in detail [39]. Moving forward, it will be interesting to examine the spectrum of mutations giving rise to ciliopathy-associated coloboma: animal models may allow dissection of tissue-specific effects of different cilia perturbations and may reveal how these crucial subcellular structures and signaling pathways may be co-opted in distinct ways at different times in development.

## Supporting information

S1 File (ZIP)
